# Supplementary material for: Identifying attacks in the Russia–Ukraine conflict using seismic array data
Source: Nature. 2023 Aug 30;621(7980):767–72. doi: 10.1038/s41586-023-06416-7 (PMC10533404; doi:10.1038/s41586-023-06416-7)
Supplement: Supplementary file 1 — Collection of point spread functions. Point spread functions showing the theoretical location uncertainty for five different locations. For each location, we show three results: when only the P-wave is present, when only the S-wave is present and when both the P-wave and the S-wave are present. [file 41586_2023_6416_MOESM1_ESM.pdf]

nature portfolio

https://doi.org/10.1038/s41586-023-06416-7

Supplementary information

Identifying attacks in the Russia–Ukraine conflict using seismic array data

In the format provided by the authors and unedited

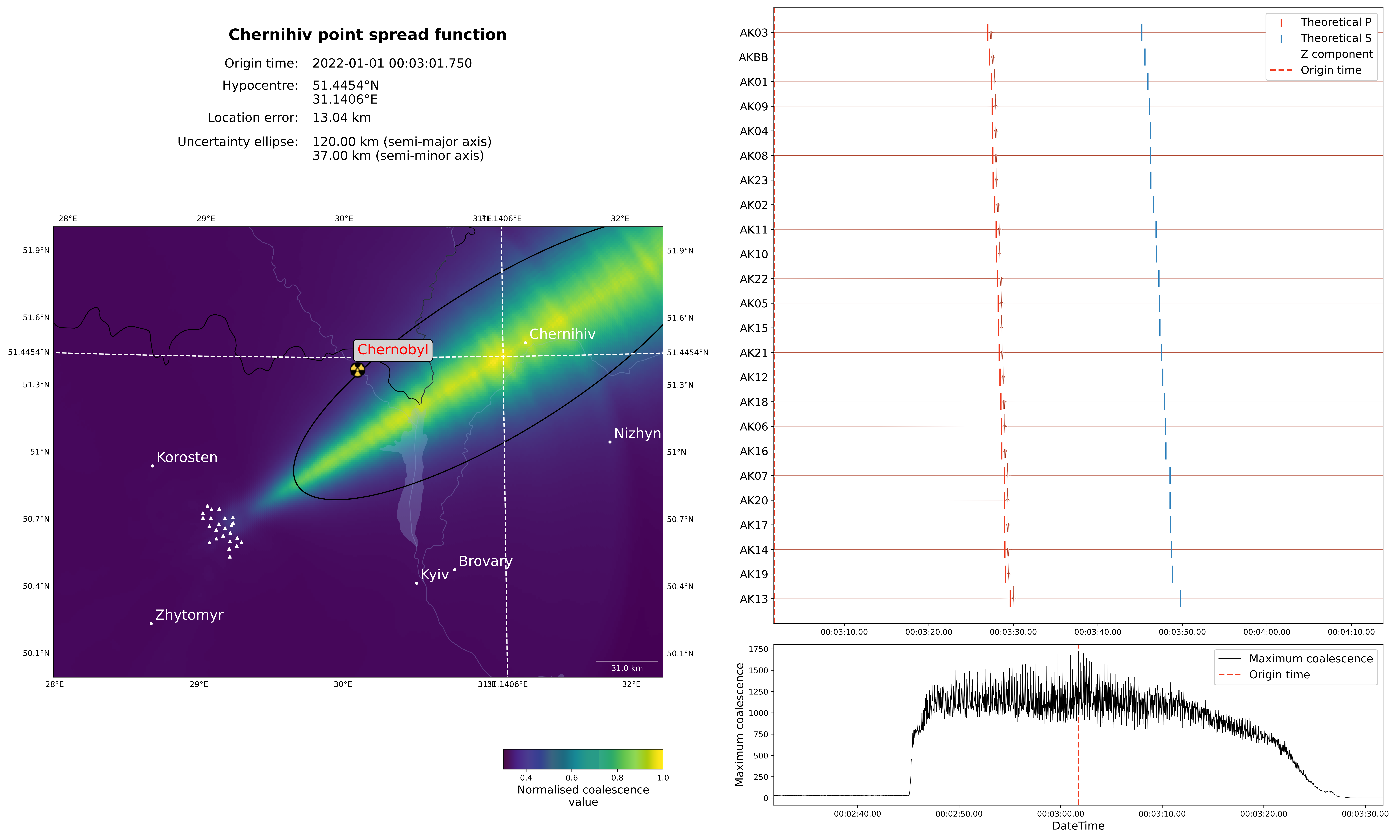
Chernihiv point spread function
Origin time: 2022-01-01 00:03:01.750
Hypocentre: 51.4454°N
31.1406°E
Location error: 13.04 km
Uncertainty ellipse: 120.00 km (semi-major axis)
37.00 km (semi-minor axis)
28°E
29°E
30°E
31.1406°E
32°E
51.9°N
51.6°N
51.4454°N
51.3°N
51°N
50.7°N
50.4°N
50.1°N
Chernobyl
Chernihiv
Nizhyn
Korosten
Brovary
Kyiv
Zhytomyr
31.0 km
0.4
0.6
0.8
1.0
Normalised coalescence value
AK03
AKBB
AK01
AK09
AK04
AK08
AK23
AK02
AK11
AK10
AK22
AK05
AK15
AK21
AK12
AK18
AK06
AK16
AK07
AK20
AK17
AK14
AK19
AK13
Theoretical P
Theoretical S
Z component
Origin time
00:03:10.00
00:03:20.00
00:03:30.00
00:03:40.00
00:03:50.00
00:04:00.00
00:04:10.00
Maximum coalescence
0
250
500
750
1000
1250
1500
1750
Maximum coalescence
Origin time
00:02:40.00
00:02:50.00
00:03:00.00
00:03:10.00
00:03:20.00
00:03:30.00
DateTime

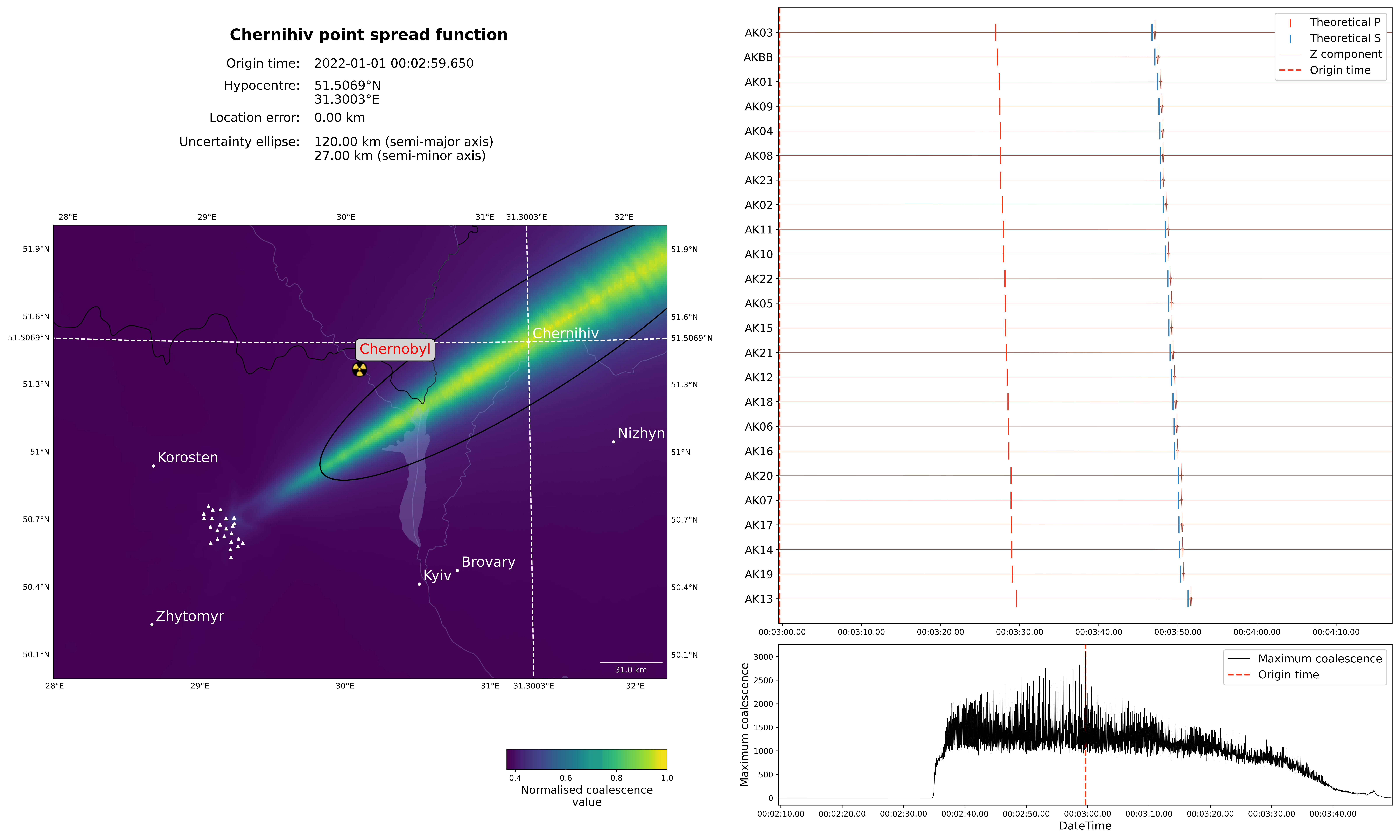

Chernihiv point spread function
Origin time: 2022-01-01 00:02:59.650
Hypocentre: 51.5069°N 31.3003°E
Location error: 0.00 km
Uncertainty ellipse: 120.00 km (semi-major axis) 27.00 km (semi-minor axis)
Chernobyl
Chernihiv
Nizhyn
Korosten
Brovary
Kyiv
Zhytomyr
31.0 km
28°E
29°E
30°E
31°E
31.3003°E
32°E
51.9°N
51.6°N
51.5069°N
51.3°N
51°N
50.7°N
50.4°N
50.1°N
0.4
0.6
0.8
1.0
Normalised coalescence value
AK03
AKBB
AK01
AK09
AK04
AK08
AK23
AK02
AK11
AK10
AK22
AK05
AK15
AK21
AK12
AK18
AK06
AK16
AK20
AK07
AK17
AK14
AK19
AK13
Theoretical P
Theoretical S
Z component
Origin time
00:03:00.00
00:03:10.00
00:03:20.00
00:03:30.00
00:03:40.00
00:03:50.00
00:04:00.00
00:04:10.00
Maximum coalescence
Origin time
Maximum coalescence
0
500
1000
1500
2000
2500
3000
00:02:10.00
00:02:20.00
00:02:30.00
00:02:40.00
00:02:50.00
00:03:00.00
00:03:10.00
00:03:20.00
00:03:30.00
00:03:40.00
DateTime

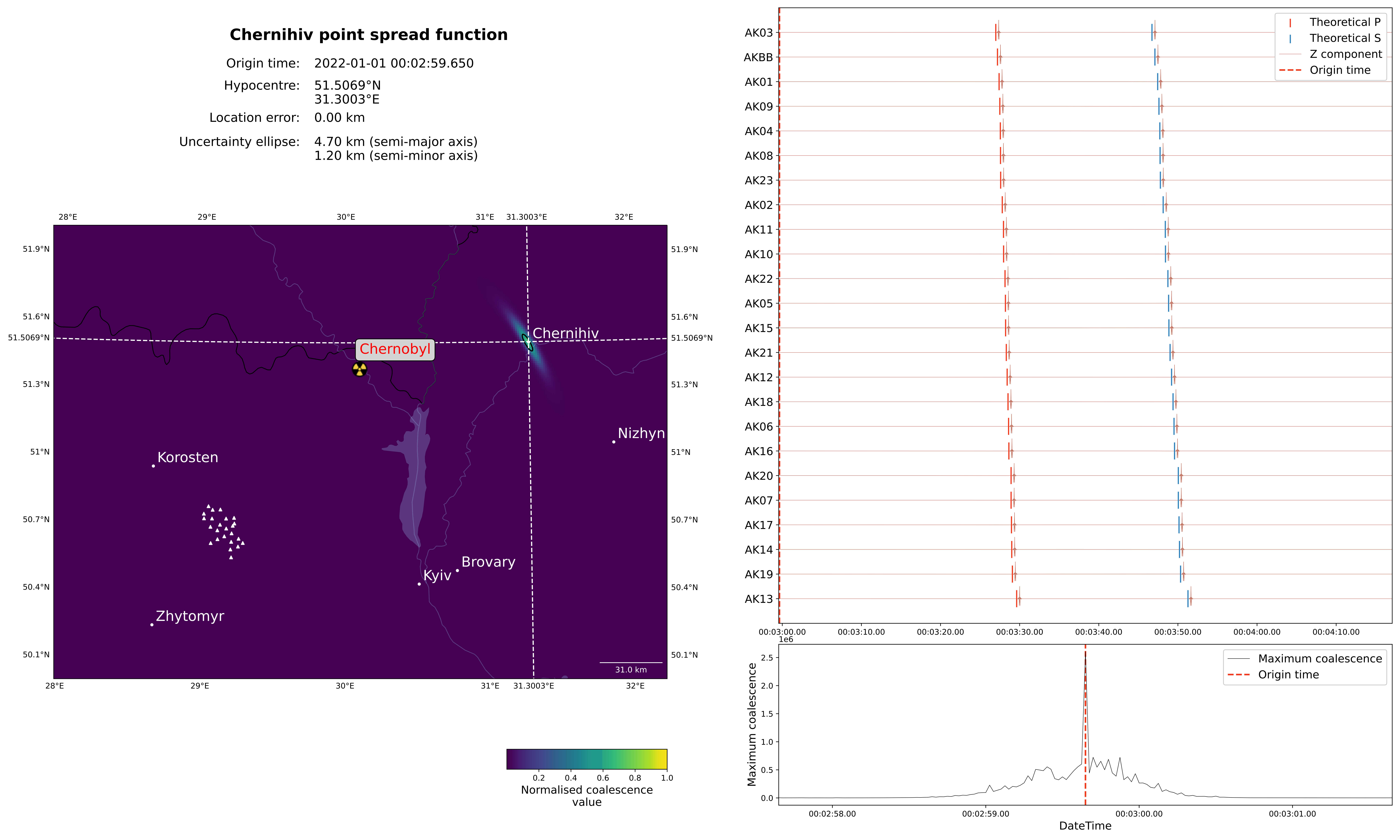
Chernihiv point spread function
Origin time: 2022-01-01 00:02:59.650
Hypocentre: 51.5069°N 31.3003°E
Location error: 0.00 km
Uncertainty ellipse: 4.70 km (semi-major axis) 1.20 km (semi-minor axis)
28°E 29°E 30°E 31°E 31.3003°E 32°E
51.9°N 51.6°N 51.5069°N 51.3°N 51°N 50.7°N 50.4°N 50.1°N
Chernihiv
Chernobyl
Nizhyn
Korosten
Brovary
Kyiv
Zhytomyr
31.0 km
Normalised coalescence value
0.2 0.4 0.6 0.8 1.0
AK03 AKBB AK01 AK09 AK04 AK08 AK23 AK02 AK11 AK10 AK22 AK05 AK15 AK21 AK12 AK18 AK06 AK16 AK20 AK07 AK17 AK14 AK19 AK13
Theoretical P
Theoretical S
Z component
Origin time
00:03:00.00 00:03:10.00 00:03:20.00 00:03:30.00 00:03:40.00 00:03:50.00 00:04:00.00 00:04:10.00
1e6
Maximum coalescence
Maximum coalescence
Origin time
0.0 0.5 1.0 1.5 2.0 2.5
00:02:58.00 00:02:59.00 00:03:00.00 00:03:01.00
DateTime

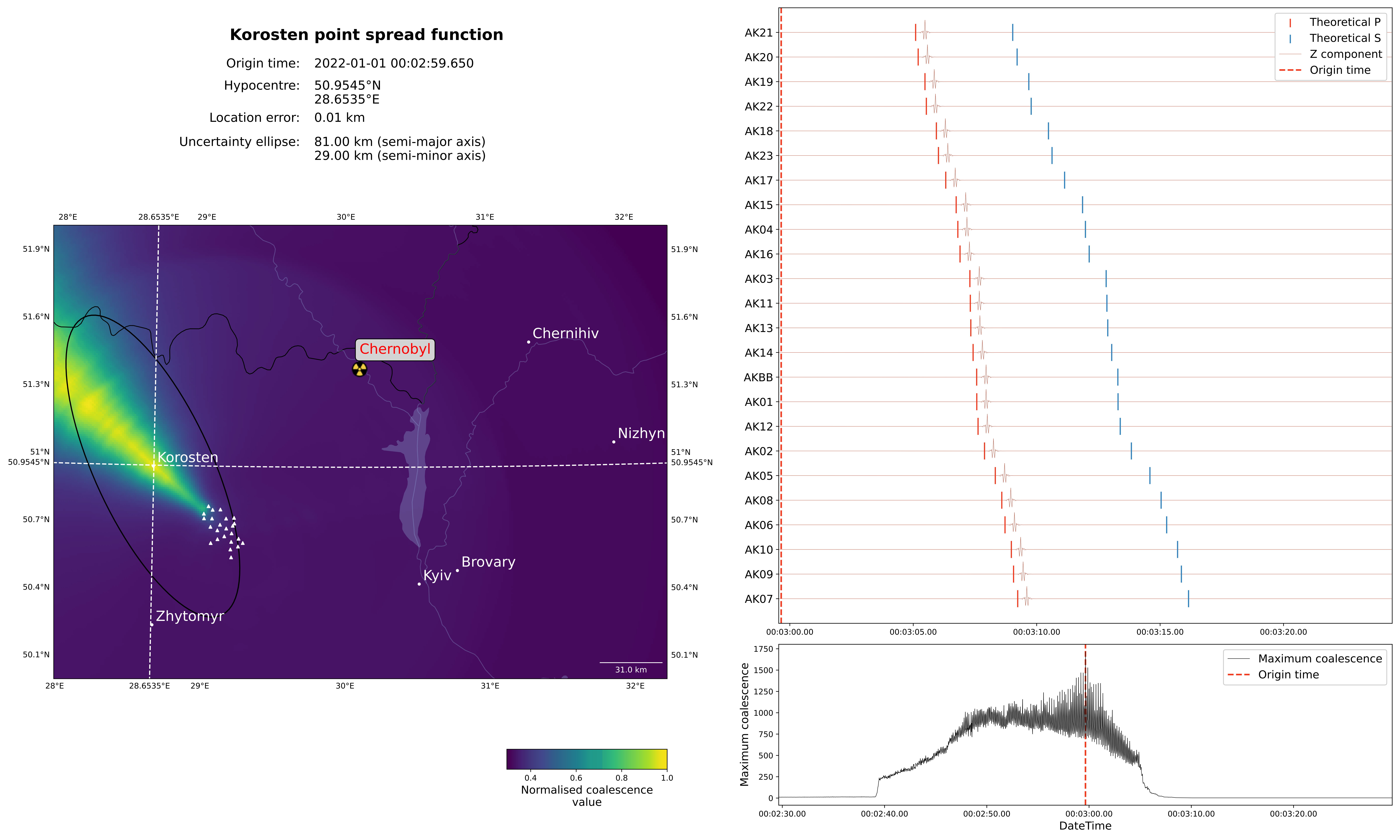

Korosten point spread function
Origin time: 2022-01-01 00:02:59.650
Hypocentre: 50.9545°N 28.6535°E
Location error: 0.01 km
Uncertainty ellipse: 81.00 km (semi-major axis) 29.00 km (semi-minor axis)
28°E
28.6535°E
29°E
30°E
31°E
32°E
51.9°N
51.6°N
51.3°N
51°N
50.9545°N
50.7°N
50.4°N
50.1°N
Chernobyl
Chernihiv
Nizhyn
Korosten
Brovary
Kyiv
Zhytomyr
31.0 km
0.4
0.6
0.8
1.0
Normalised coalescence value
AK21
AK20
AK19
AK22
AK18
AK23
AK17
AK15
AK04
AK16
AK03
AK11
AK13
AK14
AKBB
AK01
AK12
AK02
AK05
AK08
AK06
AK10
AK09
AK07
Theoretical P
Theoretical S
Z component
Origin time
00:03:00.00
00:03:05.00
00:03:10.00
00:03:15.00
00:03:20.00
Maximum coalescence
Origin time
Maximum coalescence
1750
1500
1250
1000
750
500
250
0
00:02:30.00
00:02:40.00
00:02:50.00
00:03:00.00
00:03:10.00
00:03:20.00
DateTime

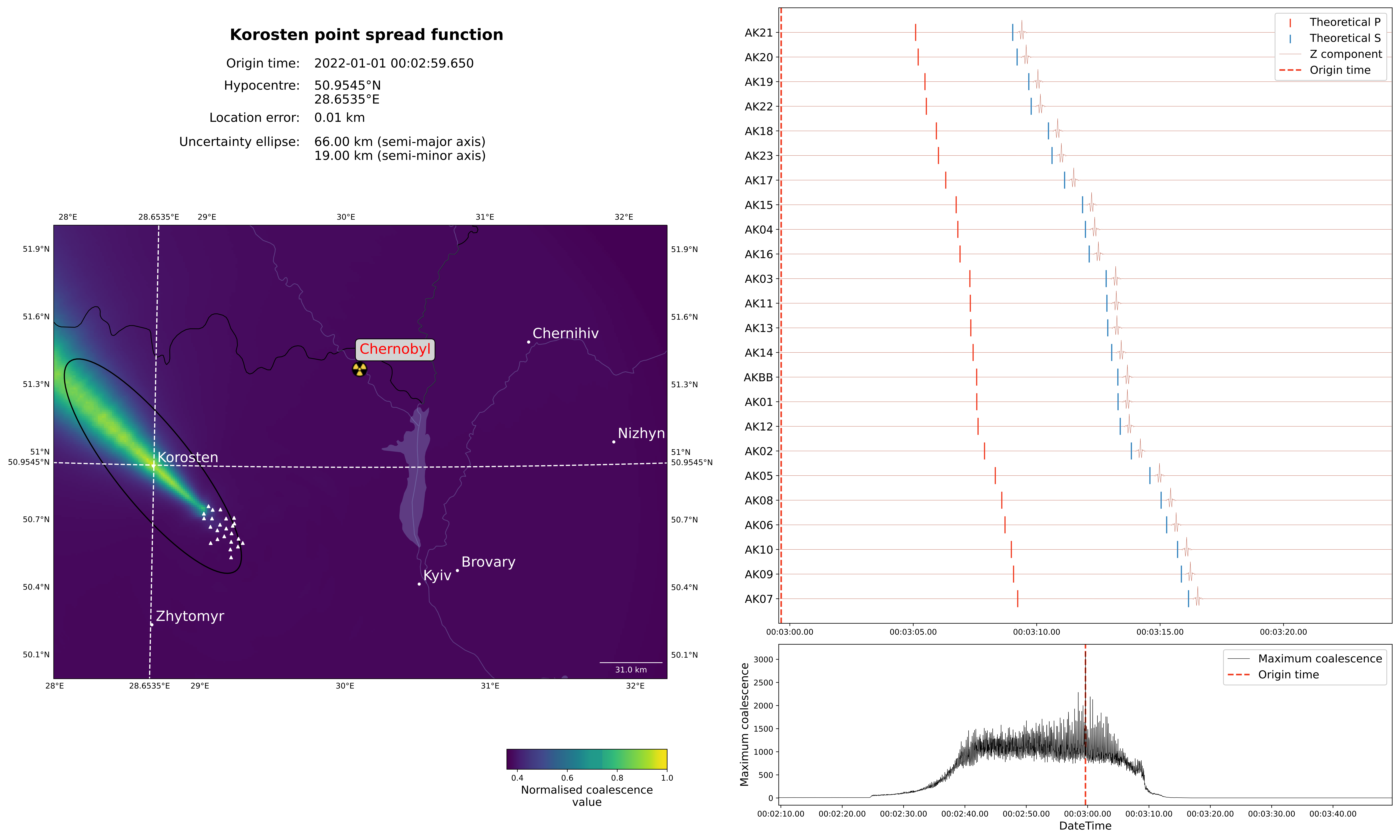
Korosten point spread function
Origin time: 2022-01-01 00:02:59.650
Hypocentre: 50.9545°N 28.6535°E
Location error: 0.01 km
Uncertainty ellipse: 66.00 km (semi-major axis) 19.00 km (semi-minor axis)
Chernobyl
Chernihiv
Nizhyn
Korosten
Brovary
Kyiv
Zhytomyr
31.0 km
28°E
28.6535°E
29°E
30°E
31°E
32°E
51.9°N
51.6°N
51.3°N
51°N
50.9545°N
50.7°N
50.4°N
50.1°N
Normalised coalescence value
0.4
0.6
0.8
1.0
Theoretical P
Theoretical S
Z component
Origin time
AK21
AK20
AK19
AK22
AK18
AK23
AK17
AK15
AK04
AK16
AK03
AK11
AK13
AK14
AKBB
AK01
AK12
AK02
AK05
AK08
AK06
AK10
AK09
AK07
00:03:00.00
00:03:05.00
00:03:10.00
00:03:15.00
00:03:20.00
Maximum coalescence
Origin time
Maximum coalescence
0
500
1000
1500
2000
2500
3000
00:02:10.00
00:02:20.00
00:02:30.00
00:02:40.00
00:02:50.00
00:03:00.00
00:03:10.00
00:03:20.00
00:03:30.00
00:03:40.00
DateTime

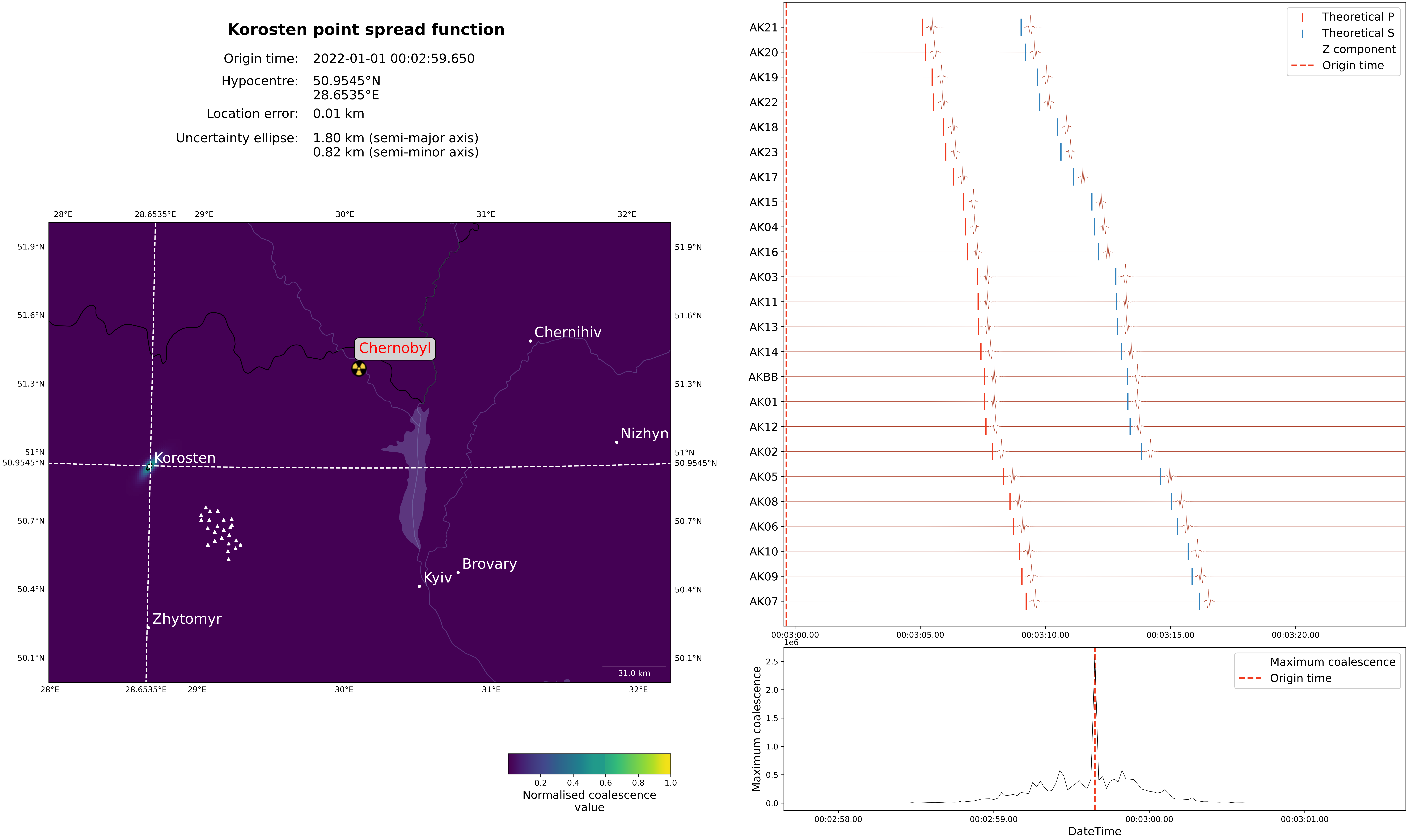

Korosten point spread function
Origin time: 2022-01-01 00:02:59.650
Hypocentre: 50.9545°N 28.6535°E
Location error: 0.01 km
Uncertainty ellipse: 1.80 km (semi-major axis) 0.82 km (semi-minor axis)
Chernobyl
Chernihiv
Nizhyn
Korosten
Brovary
Kyiv
Zhytomyr
31.0 km
Normalised coalescence value
Theoretical P
Theoretical S
Z component
Origin time
Maximum coalescence
Maximum coalescence
DateTime

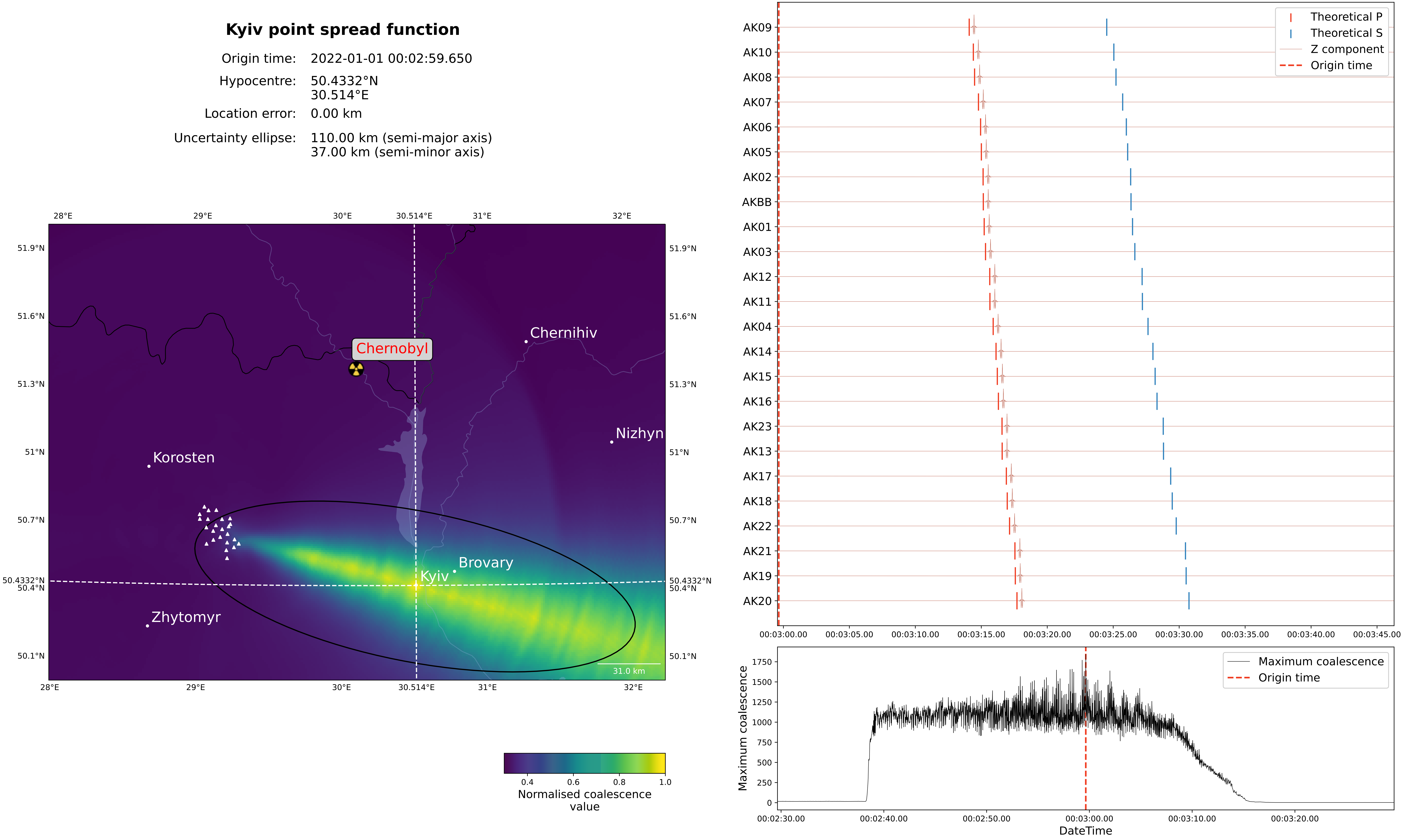

Kyiv point spread function
Origin time: 2022-01-01 00:02:59.650
Hypocentre: 50.4332°N 30.514°E
Location error: 0.00 km
Uncertainty ellipse: 110.00 km (semi-major axis) 37.00 km (semi-minor axis)
Chernobyl
Chernihiv
Nizhyn
Korosten
Brovary
Kyiv
Zhytomyr
31.0 km
Normalised coalescence value
Theoretical P
Theoretical S
Z component
Origin time
Maximum coalescence
DateTime

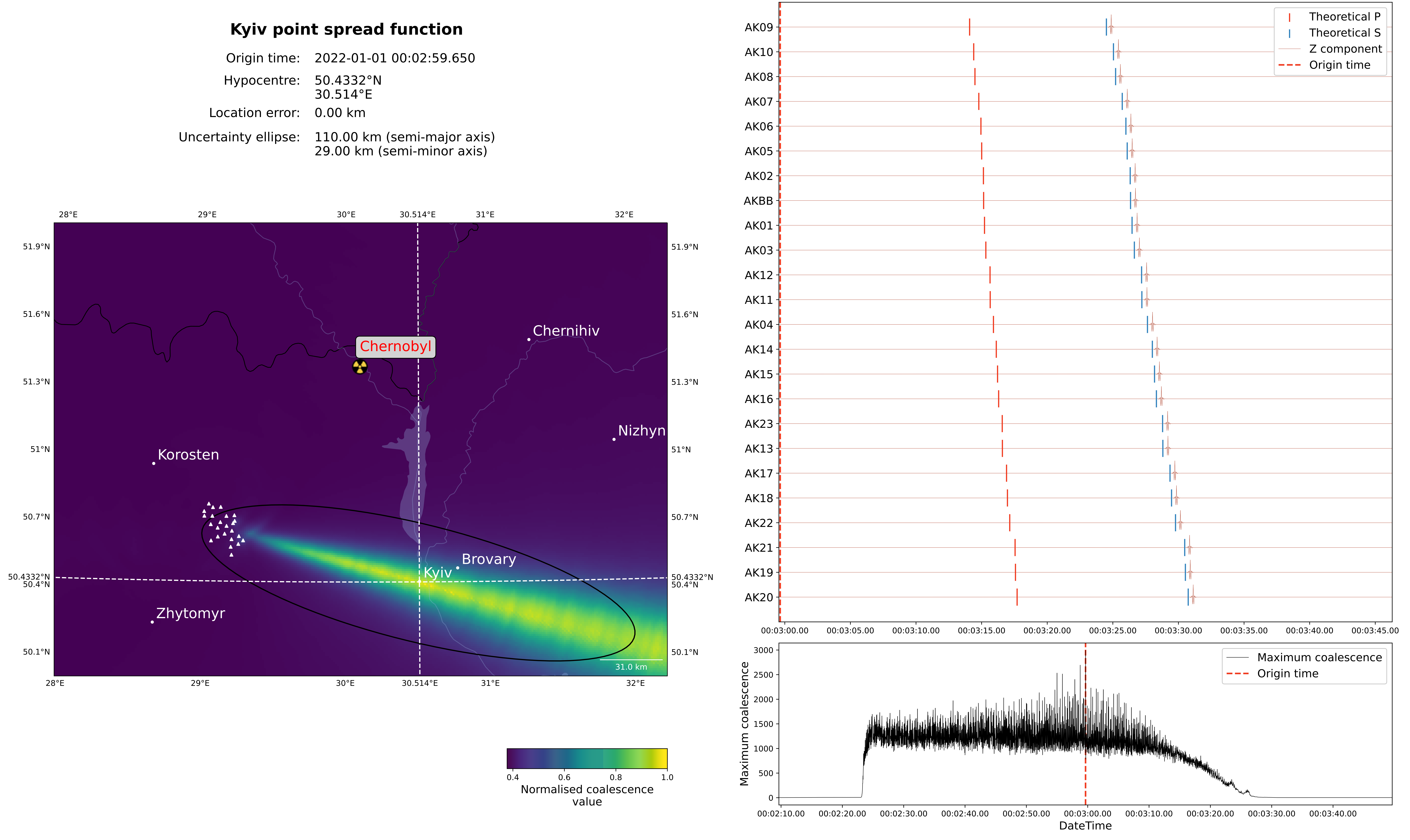

Kyiv point spread function
Origin time: 2022-01-01 00:02:59.650
Hypocentre: 50.4332°N 30.514°E
Location error: 0.00 km
Uncertainty ellipse: 110.00 km (semi-major axis) 29.00 km (semi-minor axis)
28°E 29°E 30°E 30.514°E 31°E 32°E
51.9°N 51.6°N 51.3°N 51°N 50.7°N 50.4332°N 50.4°N 50.1°N
Chernobyl
Chernihiv
Nizhyn
Korosten
Brovary
Kyiv
Zhytomyr
31.0 km
0.4 0.6 0.8 1.0
Normalised coalescence value
AK09 AK10 AK08 AK07 AK06 AK05 AK02 AKBB AK01 AK03 AK12 AK11 AK04 AK14 AK15 AK16 AK23 AK13 AK17 AK18 AK22 AK21 AK19 AK20
Theoretical P
Theoretical S
Z component
Origin time
00:03:00.00 00:03:05.00 00:03:10.00 00:03:15.00 00:03:20.00 00:03:25.00 00:03:30.00 00:03:35.00 00:03:40.00 00:03:45.00
Maximum coalescence
Origin time
Maximum coalescence
0 500 1000 1500 2000 2500 3000
00:02:10.00 00:02:20.00 00:02:30.00 00:02:40.00 00:02:50.00 00:03:00.00 00:03:10.00 00:03:20.00 00:03:30.00 00:03:40.00
DateTime

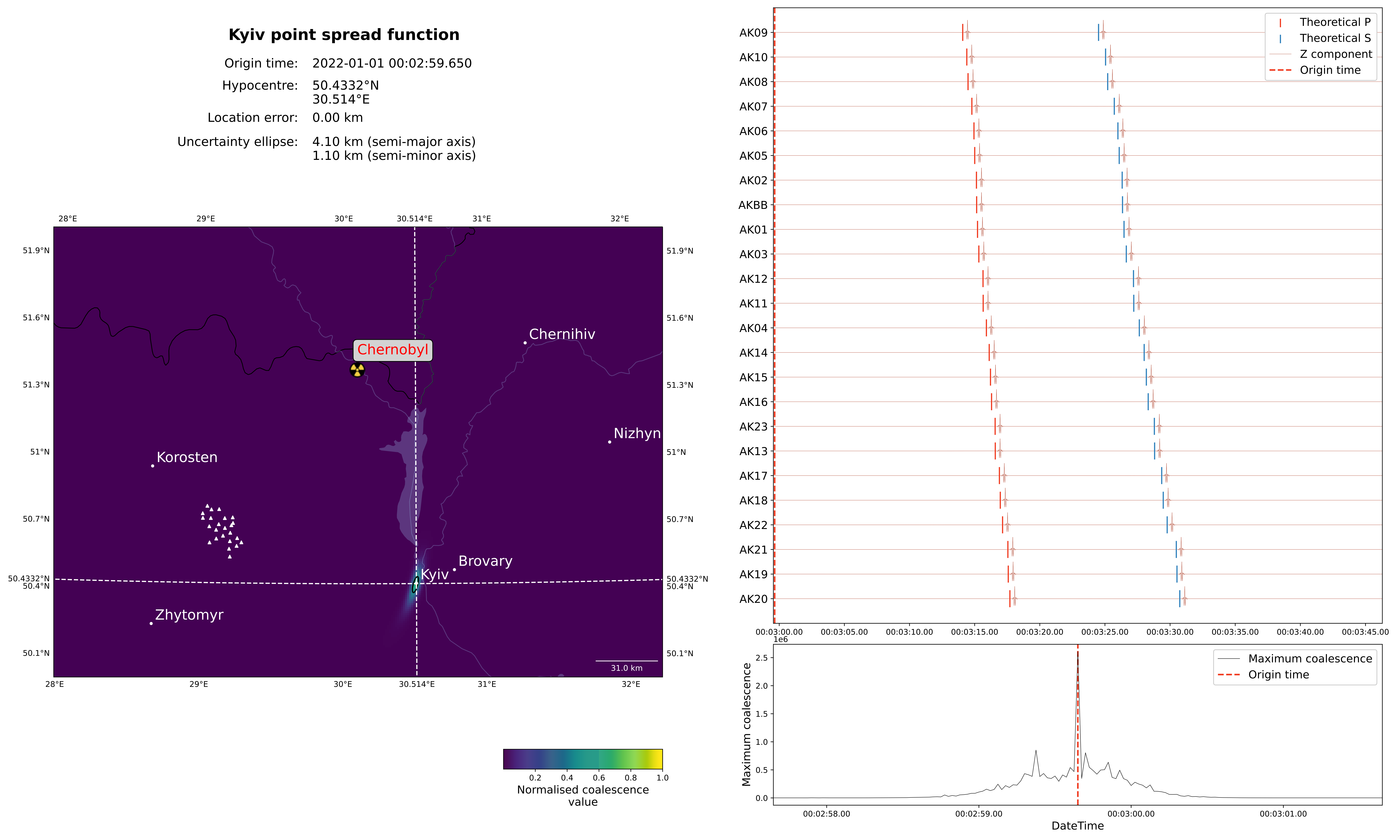

Kyiv point spread function
Origin time: 2022-01-01 00:02:59.650
Hypocentre: 50.4332°N 30.514°E
Location error: 0.00 km
Uncertainty ellipse: 4.10 km (semi-major axis) 1.10 km (semi-minor axis)
28°E
29°E
30°E
30.514°E
31°E
32°E
51.9°N
51.6°N
51.3°N
51°N
50.7°N
50.4332°N
50.4°N
50.1°N
Chernobyl
Chernihiv
Nizhyn
Korosten
Brovary
Kyiv
Zhytomyr
31.0 km
0.2
0.4
0.6
0.8
1.0
Normalised coalescence value
AK09
AK10
AK08
AK07
AK06
AK05
AK02
AKBB
AK01
AK03
AK12
AK11
AK04
AK14
AK15
AK16
AK23
AK13
AK17
AK18
AK22
AK21
AK19
AK20
Theoretical P
Theoretical S
Z component
Origin time
00:03:00.00
00:03:05.00
00:03:10.00
00:03:15.00
00:03:20.00
00:03:25.00
00:03:30.00
00:03:35.00
00:03:40.00
00:03:45.00
1e6
Maximum coalescence
0.0
0.5
1.0
1.5
2.0
2.5
Maximum coalescence
Origin time
00:02:58.00
00:02:59.00
00:03:00.00
00:03:01.00
DateTime

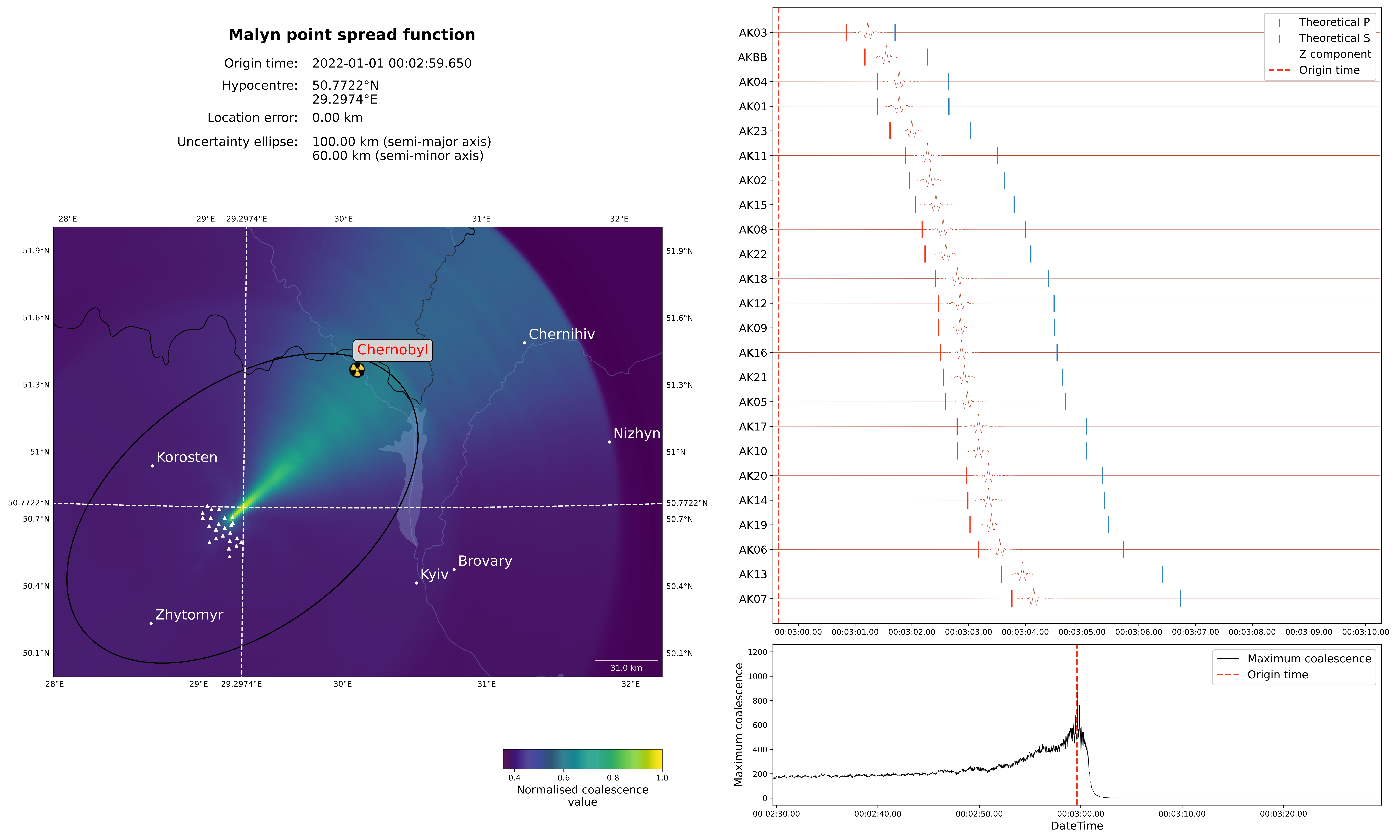

Malyn point spread function
Origin time: 2022-01-01 00:02:59.650
Hypocentre: 50.7722°N 29.2974°E
Location error: 0.00 km
Uncertainty ellipse: 100.00 km (semi-major axis) 60.00 km (semi-minor axis)
Chernobyl
Chernihiv
Nizhyn
Korosten
Brovary
Kyiv
Zhytomyr
31.0 km
Normalised coalescence value
Theoretical P
Theoretical S
Z component
Origin time
Maximum coalescence
DateTime

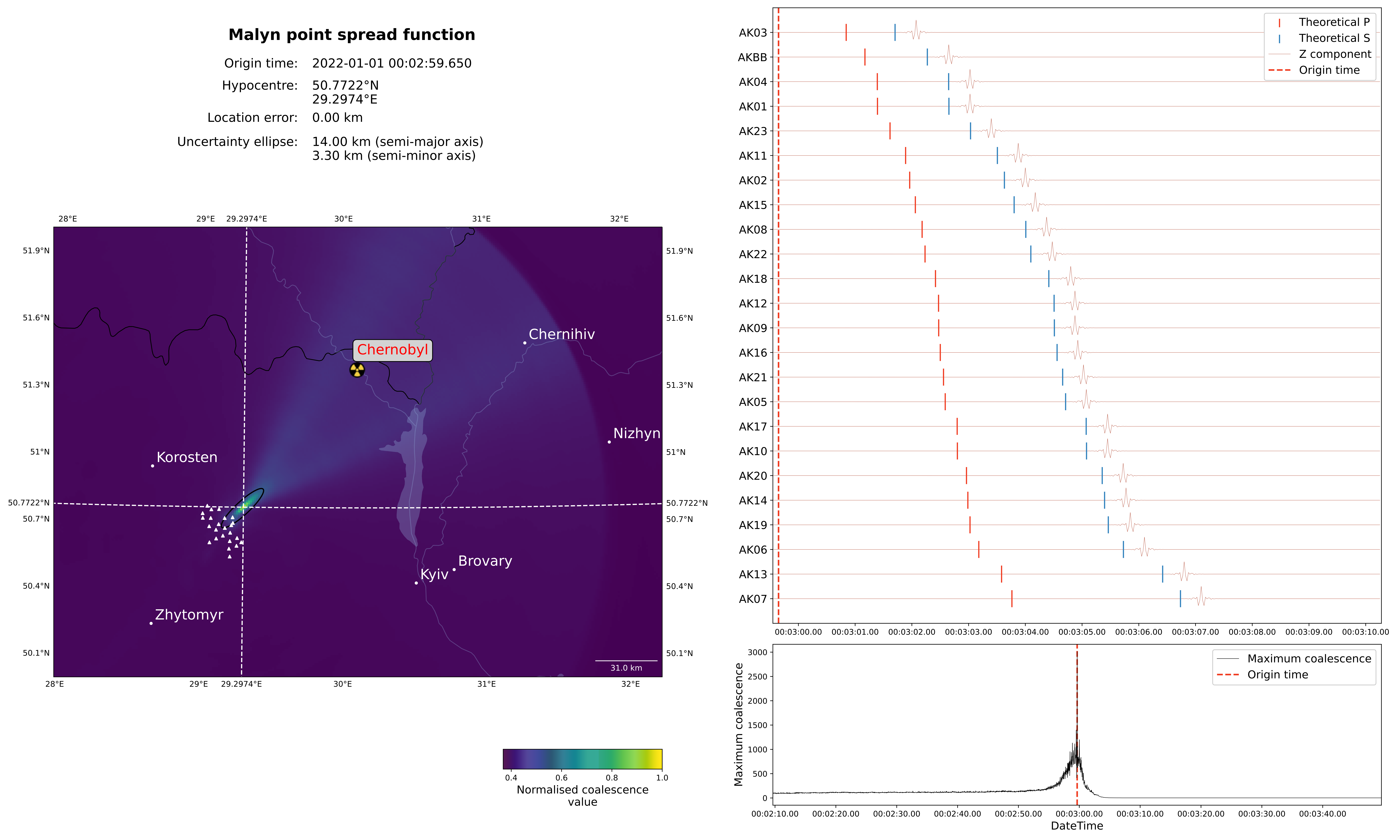
Malyn point spread function
Origin time: 2022-01-01 00:02:59.650
Hypocentre: 50.7722°N
29.2974°E
Location error: 0.00 km
Uncertainty ellipse: 14.00 km (semi-major axis)
3.30 km (semi-minor axis)
28°E
29°E
29.2974°E
30°E
31°E
32°E
51.9°N
51.6°N
51.3°N
51°N
50.7722°N
50.7°N
50.4°N
50.1°N
Chernobyl
Chernihiv
Nizhyn
Korosten
Brovary
Kyiv
Zhytomyr
31.0 km
0.4
0.6
0.8
1.0
Normalised coalescence value
AK03
AKBB
AK04
AK01
AK23
AK11
AK02
AK15
AK08
AK22
AK18
AK12
AK09
AK16
AK21
AK05
AK17
AK10
AK20
AK14
AK19
AK06
AK13
AK07
Theoretical P
Theoretical S
Z component
Origin time
00:03:00.00
00:03:01.00
00:03:02.00
00:03:03.00
00:03:04.00
00:03:05.00
00:03:06.00
00:03:07.00
00:03:08.00
00:03:09.00
00:03:10.00
Maximum coalescence
Origin time
Maximum coalescence
3000
2500
2000
1500
1000
500
0
00:02:10.00
00:02:20.00
00:02:30.00
00:02:40.00
00:02:50.00
00:03:00.00
00:03:10.00
00:03:20.00
00:03:30.00
00:03:40.00
DateTime

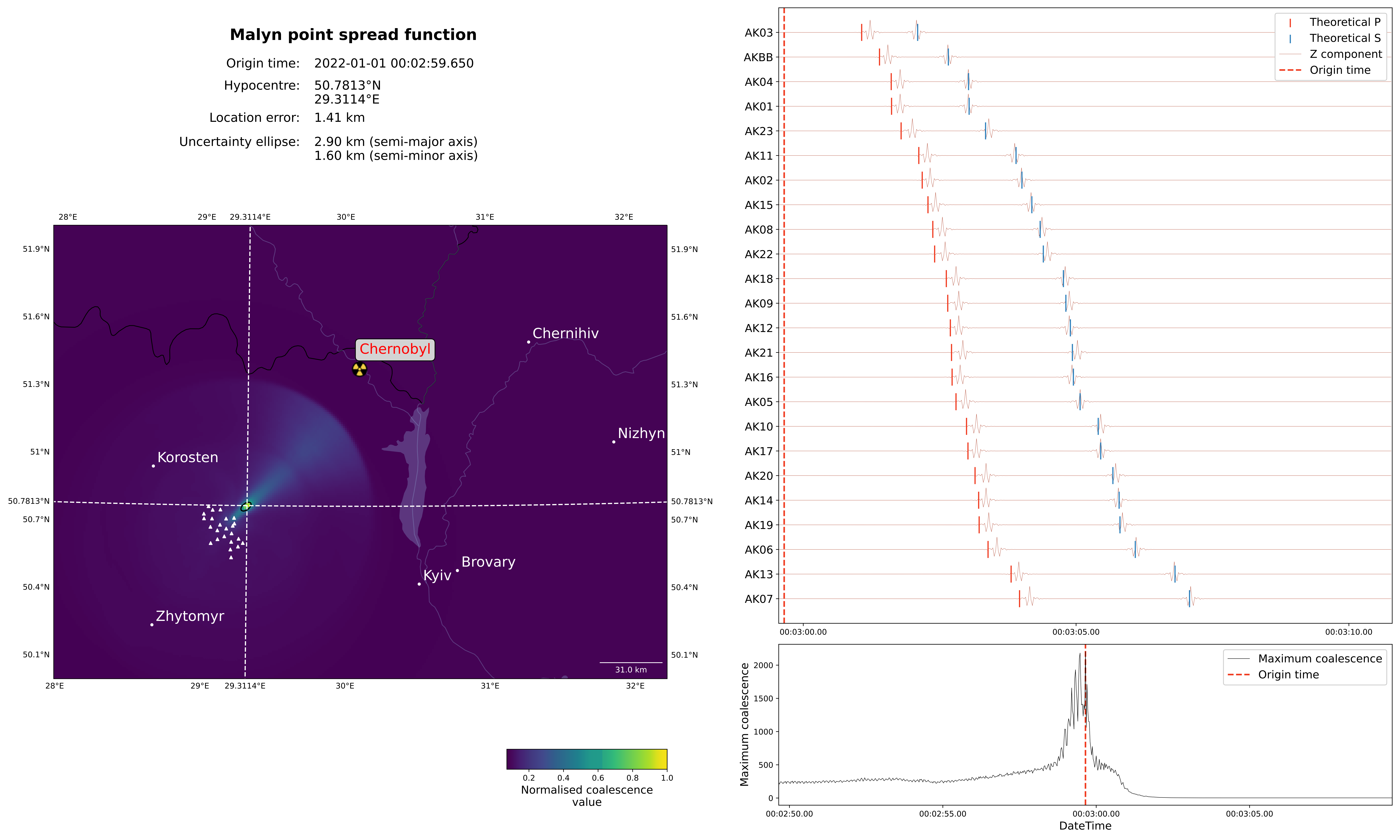

Malyn point spread function
Origin time: 2022-01-01 00:02:59.650
Hypocentre: 50.7813°N 29.3114°E
Location error: 1.41 km
Uncertainty ellipse: 2.90 km (semi-major axis) 1.60 km (semi-minor axis)
28°E
29°E
29.3114°E
30°E
31°E
32°E
51.9°N
51.6°N
51.3°N
51°N
50.7813°N
50.7°N
50.4°N
50.1°N
Chernobyl
Chernihiv
Nizhyn
Korosten
Brovary
Kyiv
Zhytomyr
31.0 km
Normalised coalescence value
0.2
0.4
0.6
0.8
1.0
Theoretical P
Theoretical S
Z component
Origin time
AK03
AKBB
AK04
AK01
AK23
AK11
AK02
AK15
AK08
AK22
AK18
AK09
AK12
AK21
AK16
AK05
AK10
AK17
AK20
AK14
AK19
AK06
AK13
AK07
00:03:00.00
00:03:05.00
00:03:10.00
Maximum coalescence
Origin time
Maximum coalescence
2000
1500
1000
500
0
00:02:50.00
00:02:55.00
00:03:00.00
00:03:05.00
DateTime

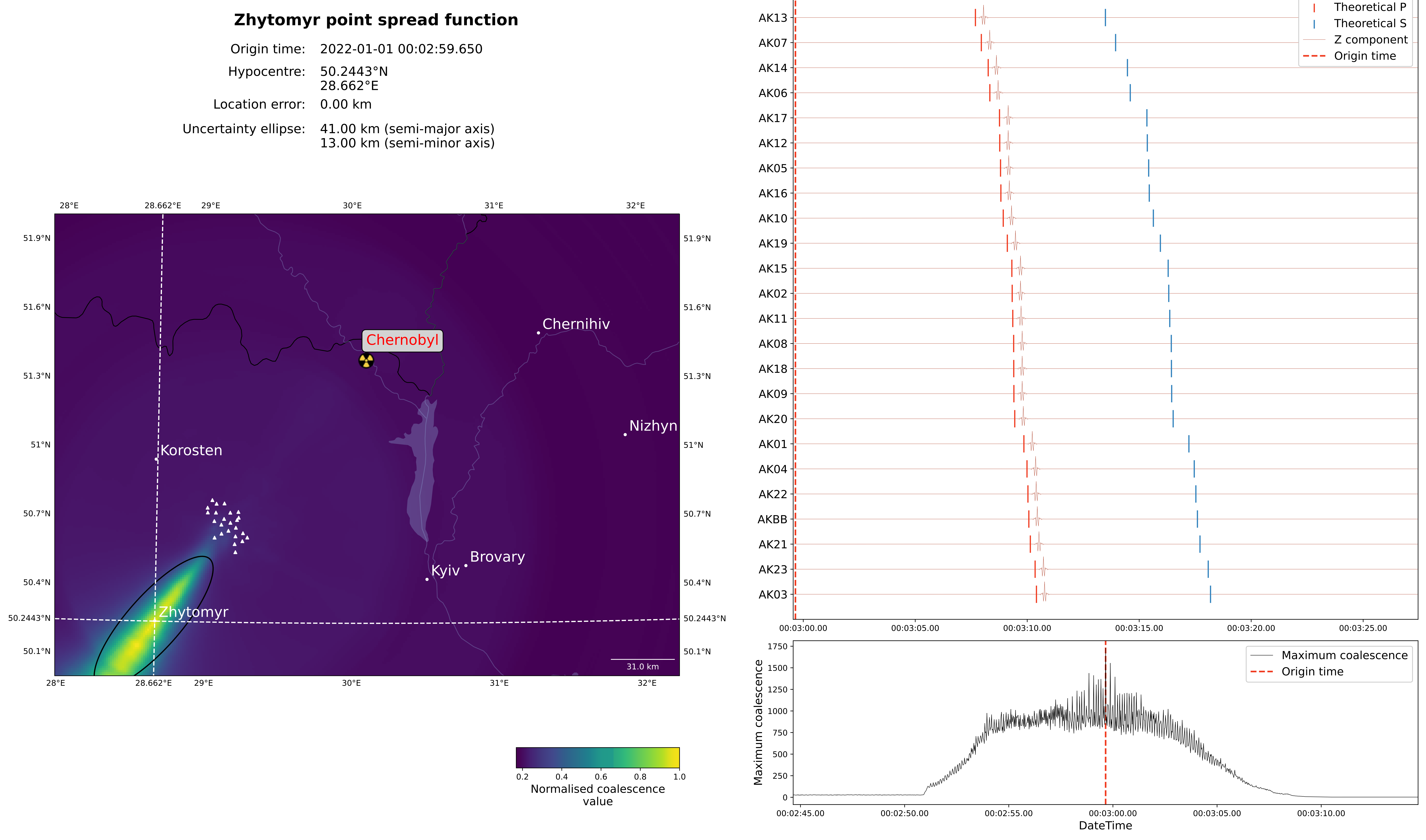

Zhytomyr point spread function
Origin time: 2022-01-01 00:02:59.650
Hypocentre: 50.2443°N 28.662°E
Location error: 0.00 km
Uncertainty ellipse: 41.00 km (semi-major axis) 13.00 km (semi-minor axis)
28°E
28.662°E
29°E
30°E
31°E
32°E
51.9°N
51.6°N
51.3°N
51°N
50.7°N
50.4°N
50.2443°N
50.1°N
Chernobyl
Chernihiv
Nizhyn
Korosten
Brovary
Kyiv
Zhytomyr
31.0 km
0.2
0.4
0.6
0.8
1.0
Normalised coalescence value
AK13
AK07
AK14
AK06
AK17
AK12
AK05
AK16
AK10
AK19
AK15
AK02
AK11
AK08
AK18
AK09
AK20
AK01
AK04
AK22
AKBB
AK21
AK23
AK03
Theoretical P
Theoretical S
Z component
Origin time
00:03:00.00
00:03:05.00
00:03:10.00
00:03:15.00
00:03:20.00
00:03:25.00
Maximum coalescence
Origin time
1750
1500
1250
1000
750
500
250
0
00:02:45.00
00:02:50.00
00:02:55.00
00:03:00.00
00:03:05.00
00:03:10.00
DateTime

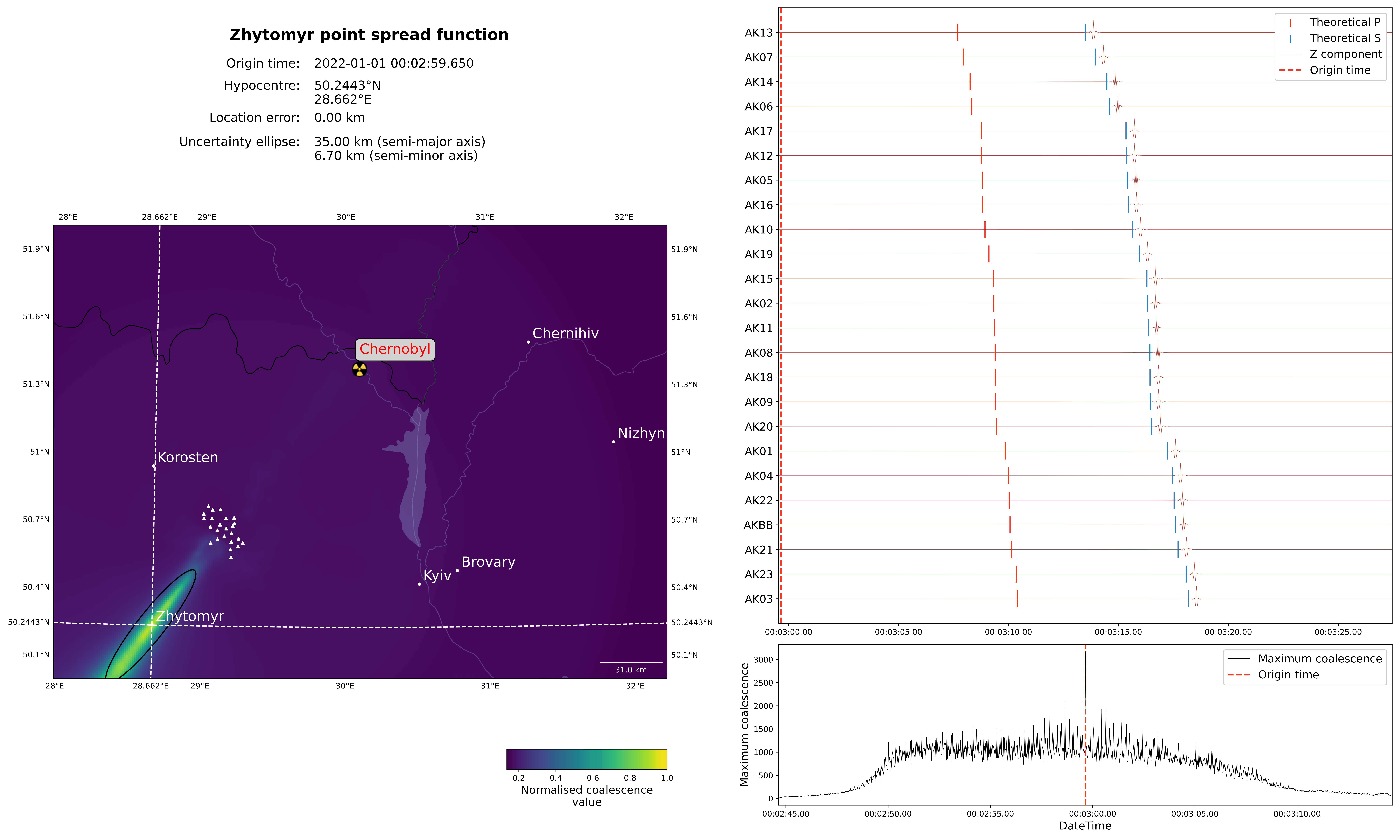
Zhytomyr point spread function
Origin time: 2022-01-01 00:02:59.650
Hypocentre: 50.2443°N 28.662°E
Location error: 0.00 km
Uncertainty ellipse: 35.00 km (semi-major axis) 6.70 km (semi-minor axis)
28°E
28.662°E
29°E
30°E
31°E
32°E
51.9°N
51.6°N
51.3°N
51°N
50.7°N
50.4°N
50.2443°N
50.1°N
Chernobyl
Chernihiv
Nizhyn
Korosten
Brovary
Kyiv
Zhytomyr
31.0 km
0.2
0.4
0.6
0.8
1.0
Normalised coalescence value
Theoretical P
Theoretical S
Z component
Origin time
AK13
AK07
AK14
AK06
AK17
AK12
AK05
AK16
AK10
AK19
AK15
AK02
AK11
AK08
AK18
AK09
AK20
AK01
AK04
AK22
AKBB
AK21
AK23
AK03
00:03:00.00
00:03:05.00
00:03:10.00
00:03:15.00
00:03:20.00
00:03:25.00
Maximum coalescence
Origin time
3000
2500
2000
1500
1000
500
0
00:02:45.00
00:02:50.00
00:02:55.00
00:03:00.00
00:03:05.00
00:03:10.00
DateTime

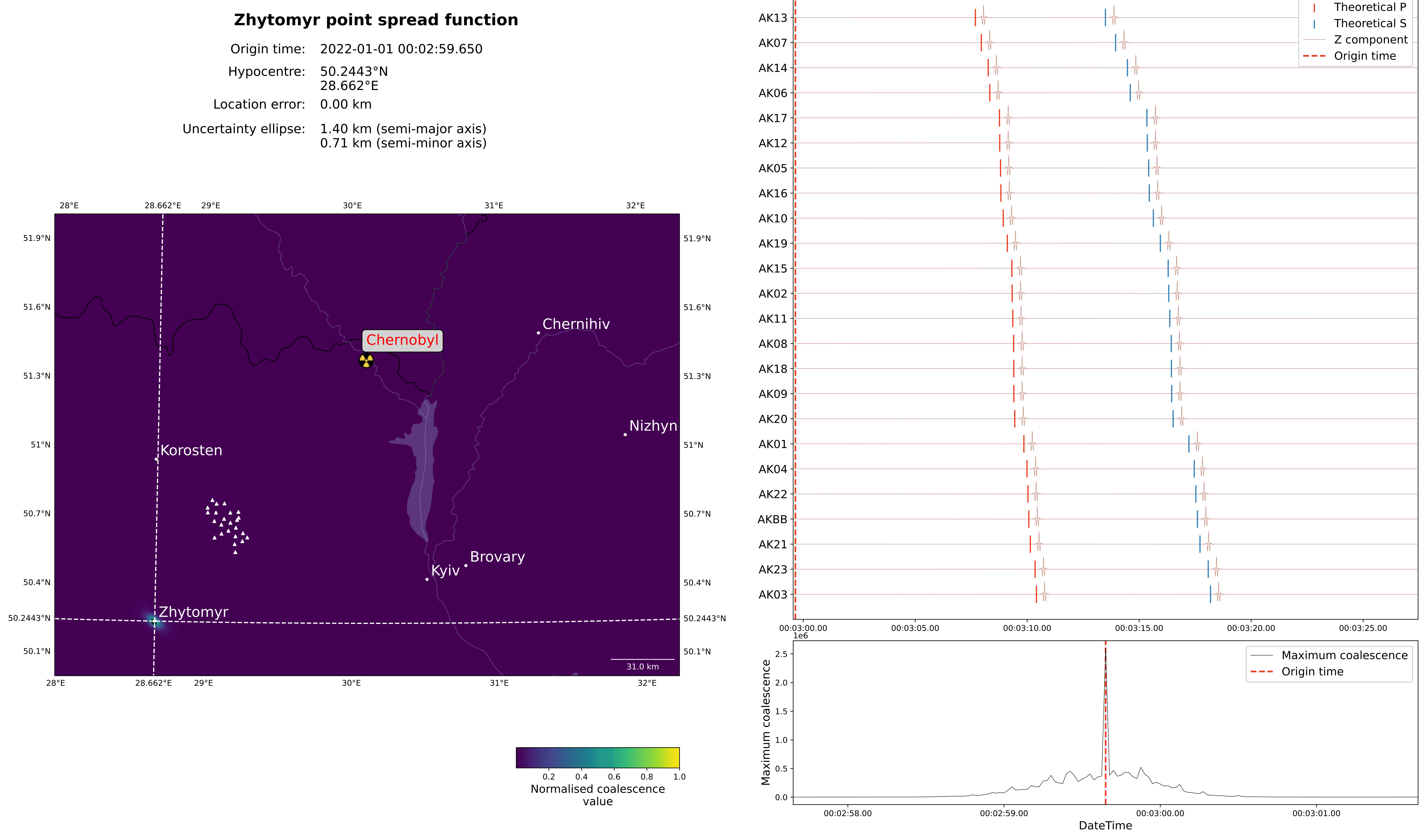
Zhytomyr point spread function
Origin time: 2022-01-01 00:02:59.650
Hypocentre: 50.2443°N 28.662°E
Location error: 0.00 km
Uncertainty ellipse: 1.40 km (semi-major axis) 0.71 km (semi-minor axis)
28°E
28.662°E
29°E
30°E
31°E
32°E
51.9°N
51.6°N
51.3°N
51°N
50.7°N
50.4°N
50.2443°N
50.1°N
Chernobyl
Chernihiv
Nizhyn
Korosten
Brovary
Kyiv
Zhytomyr
31.0 km
Normalised coalescence value
0.2
0.4
0.6
0.8
1.0
AK13
AK07
AK14
AK06
AK17
AK12
AK05
AK16
AK10
AK19
AK15
AK02
AK11
AK08
AK18
AK09
AK20
AK01
AK04
AK22
AKBB
AK21
AK23
AK03
Theoretical P
Theoretical S
Z component
Origin time
00:03:00.00
00:03:05.00
00:03:10.00
00:03:15.00
00:03:20.00
00:03:25.00
1e6
Maximum coalescence
Origin time
2.5
2.0
1.5
1.0
0.5
0.0
00:02:58.00
00:02:59.00
00:03:00.00
00:03:01.00
DateTime
